# Incidence, Surgical Treatment, and Prognosis of Anorectal Melanoma From 1973 to 2011: A Population-Based SEER Analysis

**DOI:** 10.1097/MD.0000000000002770

**Published:** 2016-02-18

**Authors:** Haiyan Chen, Yibo Cai, Yue Liu, Jinjie He, Yeting Hu, Qian Xiao, Wangxiong Hu, Kefeng Ding

**Affiliations:** From the Department of Surgical Oncology (HC, YC, YL, JH, YH, QX, KD),The Second Affiliated Hospital of Zhejiang University School of Medicine, Hangzhou ,Zhejiang, China); and Cancer Institute (Key Laboratory of Cancer Prevention and Intervention, China National Ministry of Education, Key Laboratory of Molecular Biology in Medical Sciences, Zhejiang Province) (HC, YC, YL, JH, YH, QX, WH, KD), The Second Affiliated Hospital of Zhejiang University School of Medicine, Hangzhou, Zhejiang, China.

## Abstract

Anorectal melanoma (AM) is a rare type of melanoma that accounts for 0.4% to 1.6% of total malignant melanomas. The incidence of AM increases over time, and it remains highly lethal, with a 5-year survival rate of 6% to 22%. Considering the rare nature of this disease, most studies on AM comprise isolated case reports and single-center trials, which could not provide comprehensive assessment of the disease. Therefore, we conducted a population-based study by using the Surveillance, Epidemiology, and End Results (SEER) program to provide the latest and best available evidence of AM.

We extracted all cases of AM registered in the SEER database from 1973 to 2011 (April 2014 release) and calculated age-adjusted incidence. Only cases with active follow-up were included to predict factors associated with prognosis. Survival outcomes were also compared among different types of surgery.

We identified 640 AM cases, which consisted of 265 rectal melanoma and 375 anal melanoma. The estimated annual incidence rates of AM per 1 million population were 0.259 in males and 0.407 in females, and it increased with advanced age and over time. Tumor stage and surgical treatment were independent predictors of survival. Results implied that surgery improved the prognosis of patients with local- and regional-stage AM but could not prolong the survival of patients with distant-stage AM. Moreover, the outcome of less extensive excision was not statistically different from that of more extensive excision.

This study provides an up-to-date estimation of the incidence and prognosis of AM by using SEER data. The incidence of AM continuously increases over time, despite its rarity. This disease also exhibits poor prognosis. Thus, AM must be further investigated in future studies. We also recommend surgery as the optimal treatment for local- and regional-stage AM patients but not for those with distant metastasis.

## Abstract 2

Supplemental Digital Content is available in the text

## INTRODUCTION

Melanoma is an aggressive, therapy-resistant malignancy of melanocytes. Melanoma is a major public health concern, and its incidence has continuously increased over the past 4 decades.^1,2^ Although more than 90% of melanoma has a cutaneous origin, it can also occur in mucosal, ocular, and unknown sites where melanocytes are present. Primary mucosal melanomas behave more aggressively and have poorer prognosis than cutaneous melanomas and are most common in the head and neck, anorectum, and female, with distribution of approximately 55%, 24%, and 18%, respectively.^3,4^ As the most frequent location of primary gastrointestinal tract melanoma, anorectal melanoma (AM) accounts for 0.4% to 1.6% of all malignant melanomas;^5,6^ the incidence rate of AM is about 2.7 cases per 10 million population per year in the United States.^7^ AM is likely to be unnoticed and diagnosed at an advanced stage because of its unspecific symptoms, such as bleeding, presence of a mass, and sensation of tenesmus, which are clinically consistent with benign hemorrhoid diseases.^8–10^ Only 20% to 30% of AM is located in the rectum, and the other melanomas are found within the anal canal or anal verge.^11,12^ Therapy for AM has not been standardized because of the low incidence of this disease and the lack of clinical experience. Generally, surgical excision is the primary treatment option for AM, but selection of either abdominoperineal resection or wide local excision remains controversial.^8,10,13^ Currently, AM remains a highly lethal disease, with a 5-year survival rate of 6% to 22%.^8,13^

Information on epidemiology and prognosis of AM, particularly rectal melanoma, is limited because of the rarity of this disease. Most studies in the literature include isolated case reports and single-center trials, which cannot accurately reflect the situation of AM. In this study, we provide insights into the epidemiology and survival outcomes of AM by using the Surveillance, Epidemiology, and End Results (SEER) Program. We also investigated surgical treatment for AM, particularly in terms of survival differences among different surgery types. SEER is an authoritative source of information on cancer incidence and survival in the United States; this program contains data collected from 18 cancer registries, which cover 28% of the US population.^14^ The use of this large population-based database can avoid the limitations of small size, as well as selection or treatment bias. Moreover, the results can be readily generalized and are considered more valid than institutional data because patients were treated in all types of clinical settings.^15–17^ This study aims to provide the best available evidence to help clinicians have a better understanding of AM.

## METHODS

### Ethics Statement

We got internet access to SEER database with the reference number 13504-Nov2013. And our study was approved by the Ethics Committee of the Second Affiliated Hospital of Zhejiang University School of Medicine. This observational study did not publish any information on an individual patient. Therefore, informed patient consent was not required.

### SEER Patients

**FIGURE 1:**
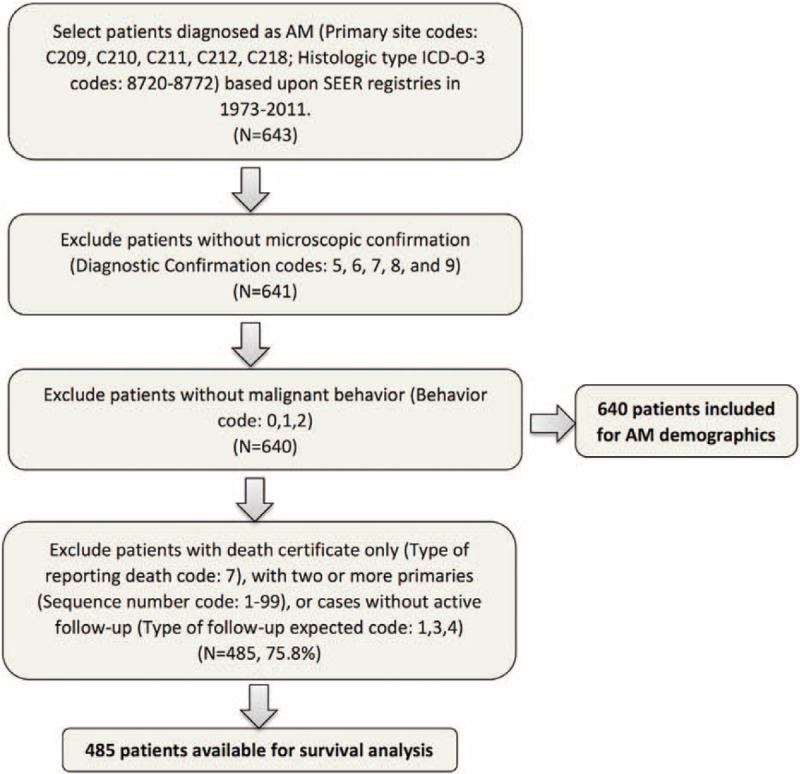
Flow chart of patient inclusion. A total of 640 AM cases were identified, and 485 of these cases were included in survival analysis.

The SEER program is the largest publicly available cancer dataset and is updated annually. The program contains data on patient demographics, tumor characteristics, first course of treatment, and follow-up information. In this research, the SEER dataset from 1973 to 2011 (April 2014 release) was used for case extraction.^18^ The included patients satisfied the following criteria: anatomic sites of rectum or anus (Primary site codes: C209, C210, C211, C212, and C218); histologically diagosed as melanoma (Histologic type ICD-O-3 codes: 8720–8772); malignant behavior (Behavior code ICD-O-3 code: 3); and microscopic confirmation of diagnosis (Diagnostic Confirmation codes: 1, 2, and 4). In the second part of the study, cases without active follow-up (Type of follow-up expected code: 1, 3, and 4) were excluded to predict factors associated with overall survival (OS) and cause-specific survival (CSS). Patients whose data were sourced solely from a death certificate (Type of Reporting Source code: 6) or those who had 2 or more primaries in their lifetime (Sequence number code: 1–99) were also excluded. The screening procedure is shown in Figure 1.

In this study, surgery types were categorized into less extensive excision (LEE) and more extensive excision (MEE) to investigate differences in survival outcomes. LEE refers to tumor resection without dissection of lymph nodes, and MEE indicates tumor resection with lymph node removal. Specifically, in rectal melanoma, cases with RX Summ Surg Prim Site (1998+) codes of 10–28 or Site specific surgery (1983–1997) codes of 10–20 are identified as LEE; by contrast, cases with Summ–Surg Prim Site (1998+) codes of 30–70 or Site specific surgery (1983–1997) codes of 30–60 are categorized as MEE. Moreover, LEE (Summ–Surg Prim Site codes: 10–27 or Site specific surgery codes: 10–40) and MEE (Summ Surg Prim Site codes: 60–63 or Site specific surgery codes: 50, 60) in anal melanoma were extracted similarly.

### Statistical Analysis

Pearson *χ*^2^ test or independent sample T test was employed to investigate significant differences between 2 groups. The incidence of AM in the United States was calculated as the number of new patients per 1 million people per year, with adjustment to the US 2000 population, and presented in terms of sex and tumor sites. Briefly, we extracted population data and calculated incidence rates by using the SEER∗Stat Version 8.2.0 software (Stata Corporation, College Station, TX). We also analyzed the incidence trends using 9 age groups (age at diagnosis: <20, 20–29, 30–39, 40–49, 50–59, 60–69, 70–79, 80–84, and ≥85-year old) and 4 observation periods (year of diagnosis: 1973–1990, 1991–2000, 2001–2005, and 2006–2011).

Univariate and multivariate models were constructed to evaluate factors correlated with survival. Survival was defined as the number of months between the date of diagnosis and the date of death of any causes (OS) or of their cancer (CSS). In the univariate model, Kaplan–Meier curves were plotted to display survival rates over time; the curves were then compared using log-rank statistics. Age at diagnosis, sex, race, stage, surgery, tumor location, and year of diagnosis were included as covariates. The multivariate Cox proportional hazards model was then fitted to estimate hazard ratios (HRs) between survival and covariates, which included variates with statistical significance in the univariate model. Cox regression method was further applied to control the influence of covariates and compare survival rates between the 2 groups. All statistical analyses were conducted using SPSS version 19.0 software (SPSS Inc, Chicago, IL). A *P* value of <0.05 was considered statistically significant. All tests were 2 sided, and confidence intervals (CIs) were set as 95%.

## RESULTS

### Characteristics of Overall Patients

A total of 640 patients were assessed as eligible for inclusion in the study by using the patient selection algorithm described in the Methods section (Figure 1); these patients included 265 (41.4%) cases with rectal melanoma and 375 (58.6%) cases with anal melanoma. The clinicopathological characteristics of the 2 groups are shown in Table 1. The mean age at diagnosis was 68.5 years (SD = 14.2), and AM was predominant in female (female/male = 1.71). Mean age (*P* = 0.662) and sex (*P* = 0.476) were not statistically different between the 2 groups. About 83.9% of the included cases were White, and more Asian patients were observed in the anal group (*P* = 0.027).

**TABLE 1:**
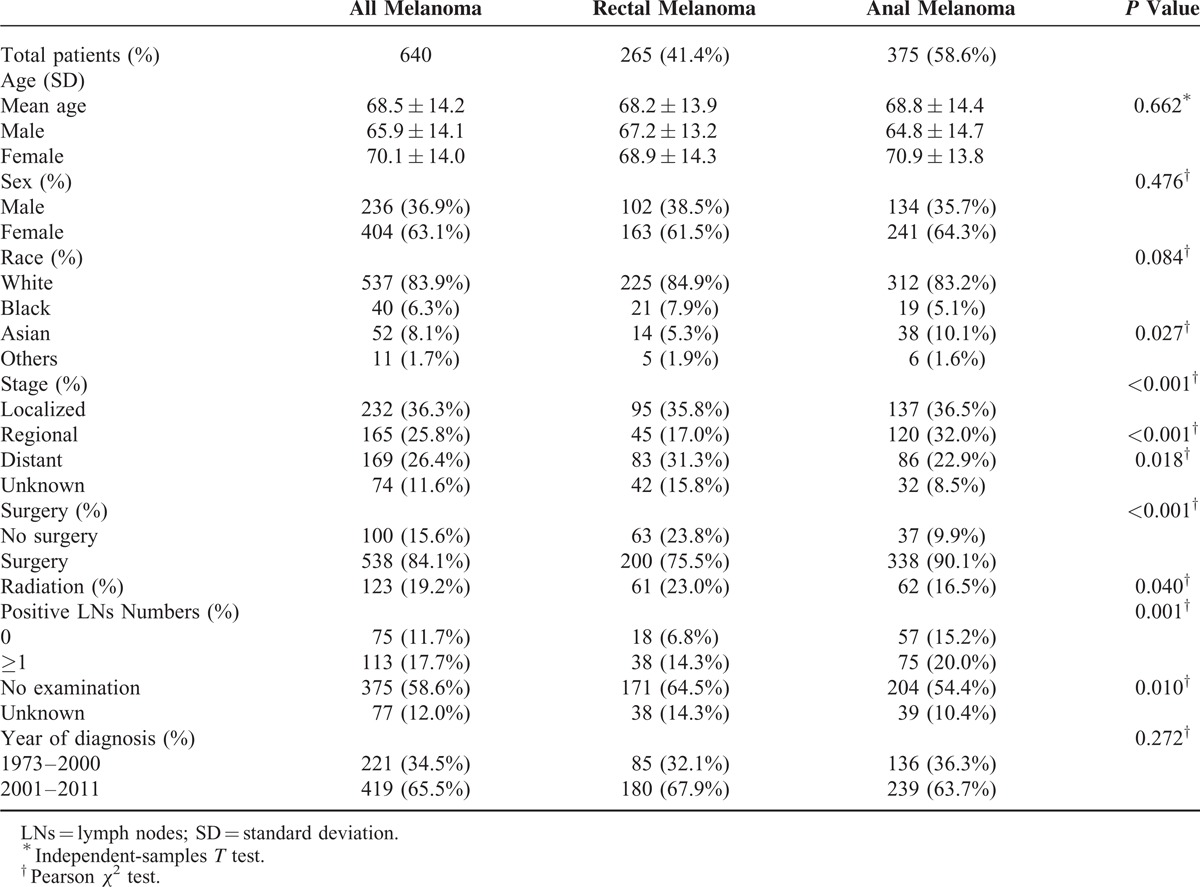
Characteristics of Patients of Anorectal Melanoma Based Upon Surveillance, Epidemiology, and End Results Registry in 1973 to 2011

In terms of stage, 232 (36.3%) patients were diagnosed at the localized stage, followed by the regional (165, 25.8%) and distant (169, 26.4%) stages. Compared with rectal melanoma, patients in the anal group were diagnosed at earlier stage, with more cases at the regional stage (*P* < 0.001) and less distant ones (*P* = 0.018). In addition, surgery was administered in 84.1% of the total patients. The number of patients with AM almost doubled from 1973–2000 (N = 221) to 2001–2011 (N = 419). Radiation and lymph node were excluded from the analysis because of the absence of corresponding data in 80.8% and 70.6% of the cases, respectively.

### Incidence

**FIGURE 2:**
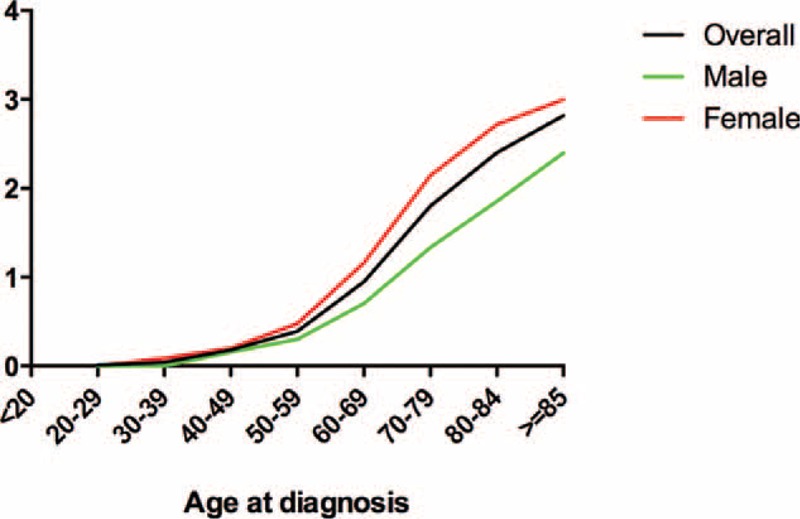
Age-adjusted incidence per 1 million population per year successively increased with advanced age in males and females.

The age-adjusted annual incidence of AM was 0.343 per 1 million population in the United States (0.259 in male and 0.407 in female). The incidence rate increased with advanced age in both sexes and at all tumor sites. Specifically, no patient with AM younger than 20 was reported, and the overall incidence rate increased from 0.013 (20–29 years) to 2.818 (≥85 years), followed by 0.000 to 2.397 and 0.026 to 3.000 for male and female, respectively (Table 2, Figure 2). Similar trend was found in anal melanoma, with incidence rates increasing from 0.006 to 2.903. However, patients with rectal melanoma exhibited the highest incidence rate at the age of 80–84 years (0.960/1,000,000). As shown in Figure S1, the incidence of anal melonoma was higher than that of rectal melanoma across almost all age groups.

**TABLE 2:**
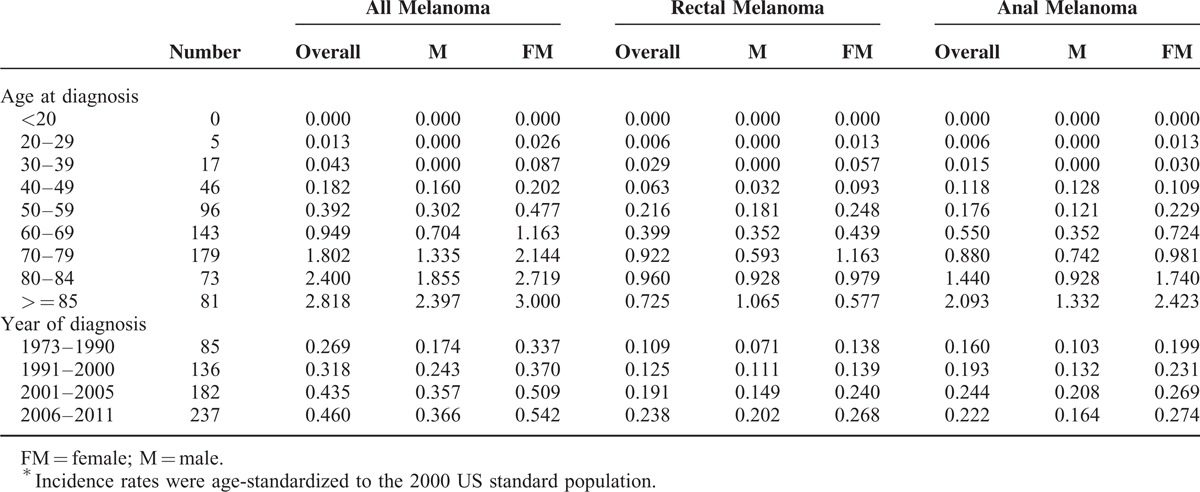
Incidence of Anorectal Melanoma per 1 Million per Year by Age and Year of Diagnosis Based Upon Surveillance, Epidemiology, and End Results Registry in 1973–2011^∗^

**FIGURE 3:**
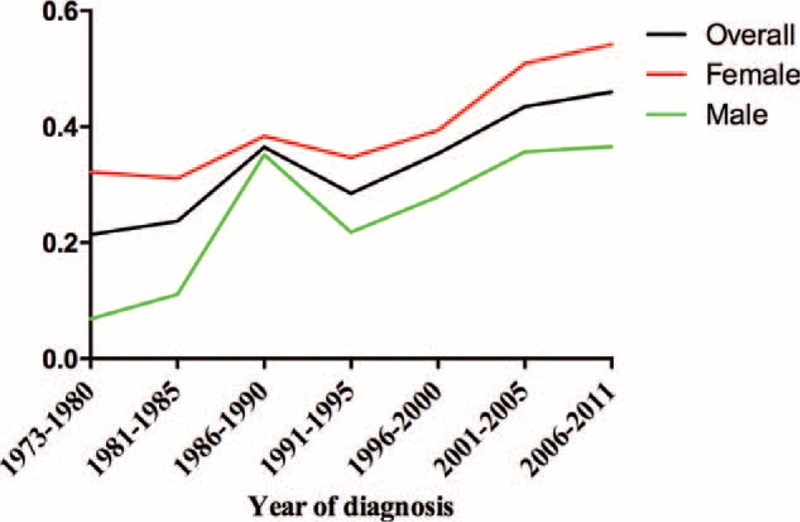
Age-adjusted incidence per 1 million population per year increased over time in males and females.

The estimated annual incidence of AM successively increased after adjusting for year of diagnosis (Table 2, Figure 3). For instance, the incidence increased from 0.269 in 1973 to 1990 to 0.460 in 2006 to 2011. Similar trend was found in male (0.174–0.366) and female (0.337–0.542) subgroups. Interestingly, the incidence of rectal melonoma consistently increased, and its increase rate (0.109–0.238) was higher than that of anal melanoma (0.160–0.222) over the study periods. Moreover, the incidence of anal melonoma minimally changed in the past 10 years and was lower than that of rectal melanoma (0.222 versus 0.238, respectively) in 2006–2011 (Figure S2).

### Potential Prognostic Factors

About 75.8% of the cases were eligible for survival analysis. Patient demographics and therapies were not different between the total patients (N = 640) and included cases (N = 485; all *P* values >0.05, Table S1). Therefore, these 485 patients were used in log-rank and Cox proportional hazard analyses to determine potential factors that affect the prognosis of AM.

As shown in Table 3 and S2, univariate models for predicting OS and CSS showed similar results. The survival closely paralleled the stage at diagnosis (*P* < 0.001 for both), and patients undergoing surgical resection exhibited improved survival (*P* < 0.001). Moreover, tumor location and year of diagnosis were related to survival.

**TABLE 3:**
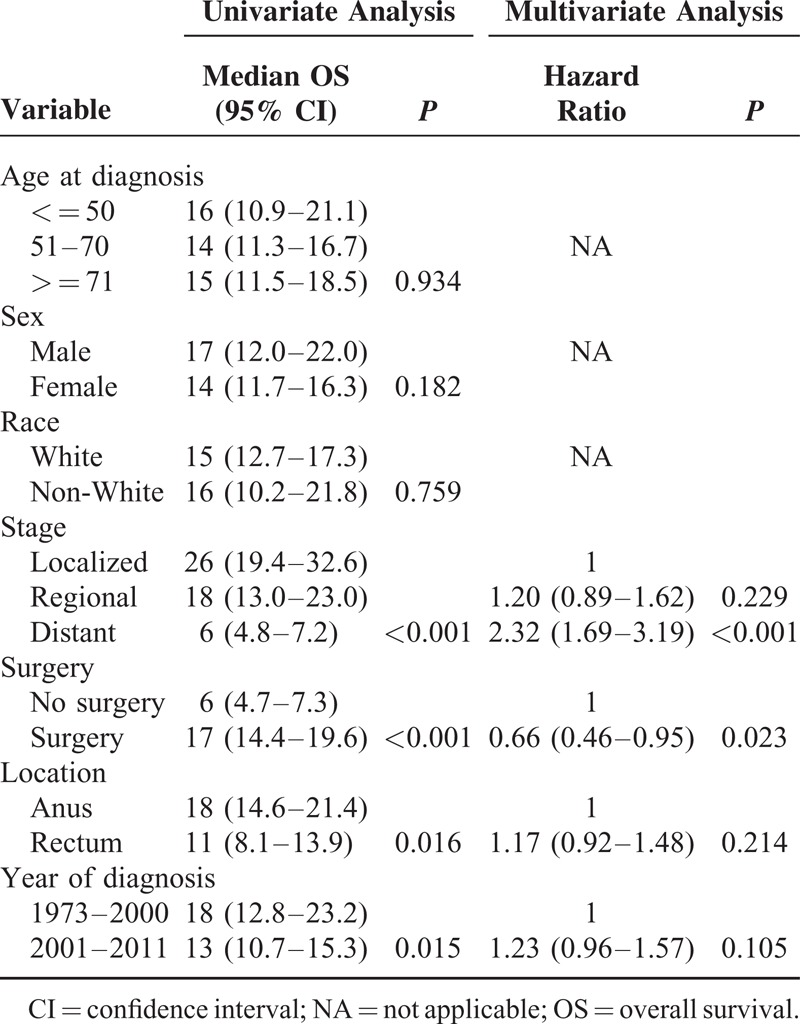
Analysis of Potential Characteristics Influencing OS

Multivariable analysis was then performed, and the results implied that patients with AM who underwent surgery showed improved prognosis, with HR = 0.66 (*P* = 0.023) and 0.69 (*P* = 0.048) in OS and CSS models, respectively (Table 3 and S2). Furthermore, distant stage predicted the worst survival (OS model: HR 2.32, *P* < 0.001; CSS model: HR 2.98, *P* < 0.001). In the CSS model, regional stage could be a risk factor for survival (HR 1.59, *P* = 0.005).

### Surgical Treatment and Survival

**FIGURE 4:**
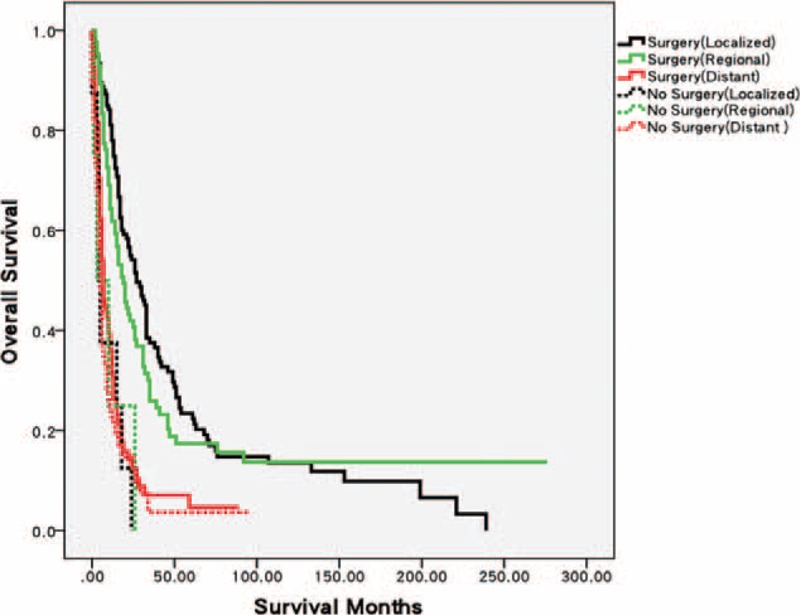
Differences in Kaplan–Meier curves of OS between surgery and no surgery groups stratified by tumor stage. Patients with AM at local or regional stage could benefit from surgery (*P* < 0.001 and *P* = 0.03). No statistical difference was observed in patients presenting distant metastasis (*P* = 0.268).

Prognosis was compared between patients receiving surgery and those without any surgical treatment. As shown in Table 4, the rate of patients who did not undergo surgery increased by 6.1% from 1973–2000 to 2001–2011. Patients undergoing surgery achieved significant survival benefits over the past 4 decades. In addition, the results of stratified analysis based on tumor stage revealed that patients at local and regional stages could benefit from surgery (*P* < 0.001 and *P* = 0.03, respectively). Conversely, survival benefits were not statistically different among patients with distant metastasis (*P* = 0.268, Figure 4). Similar results were obtained from analysis on CSS (Table S3).

**TABLE 4:**
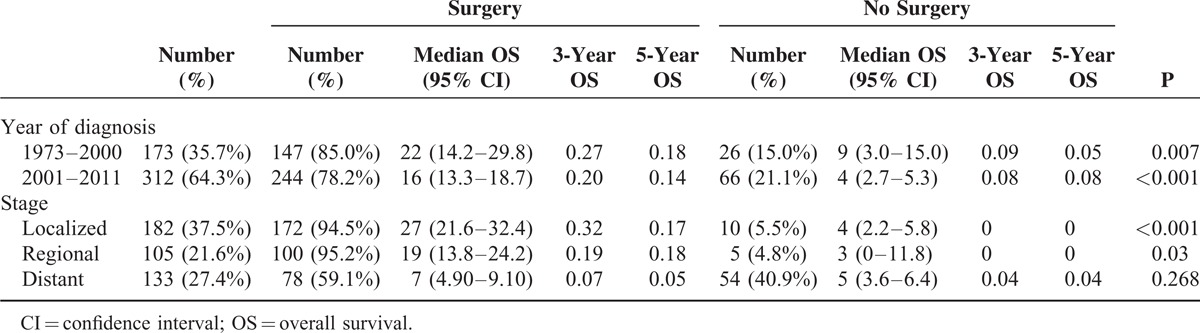
Surgical Treatment and Overall Survival by Stratified Analyses With Year of Diagnosis and Stage

Considering the differences in surgery type, we subdivided rectal and anal melanoma into 2 groups according to tumor location to investigate survival differences between LEE and MEE. No statistical differences were found in both rectal and anal groups when adjusting for tumor stage and year of diagnosis (Table 5 and S4). Interestingly, the rates of MEE decreased by 18.3% and 8.7% in rectal and anal groups, respectively, from 1973–2000 to 2001–2011.

**TABLE 5:**
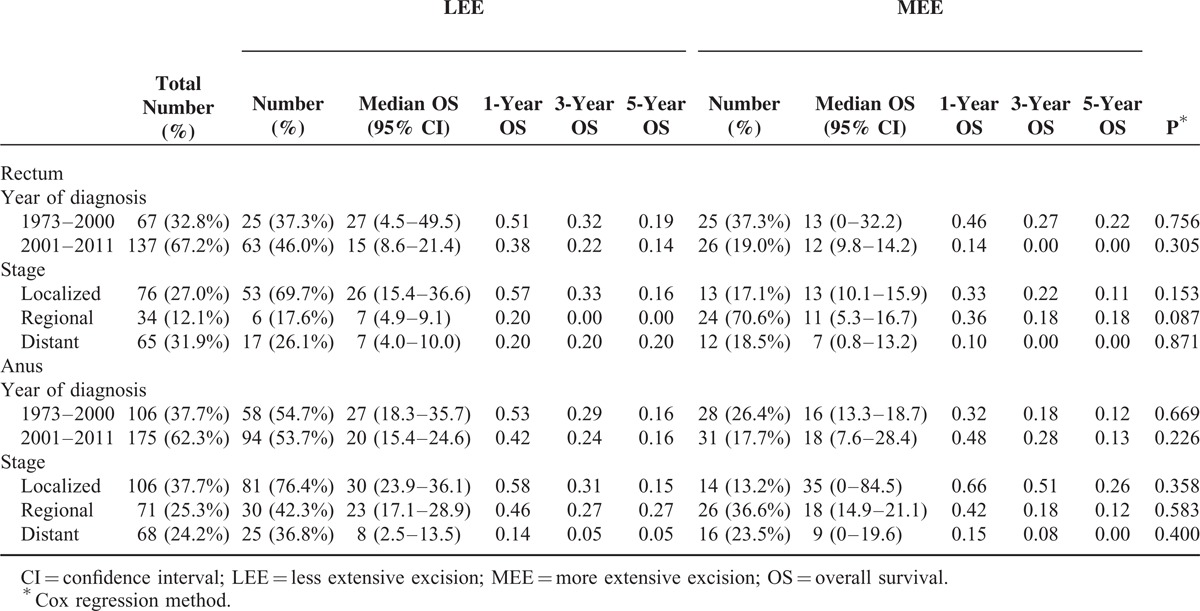
Overall Survival Differences Between Less Extensive Excision and More Extensive Excision

## DISCUSSION

In this epidemiology study, we analyzed the clinicopathological characteristics and incidence of rectal and anal melanoma by using the SEER dataset. We also estimated the survival of AM in the US population to determine potential prognostic factors. Thus far, evaluation of epidemiological features and prognosis of AM remains challenging because of the rarity of this disease. Most published studies on AM are based on experience of single institutions, and the results are heterogenous. For instance, a study at Memorial Sloan–Kettering Cancer Center reported that rectal melanoma accounts for 24% of the total AM cases,^11^ whereas Burt et al^12^ found that 35% of AM is located in the rectum. Additionally, the follow-up periods are short, and institutional data do not have sufficient power for exploring prognostic factors. To the best of our knowledge, the present research is the largest and updated population-based study of AM that consisted of 640 eligible patients from 18 cancer registries. This large population-based study can provide reliable results.

In this study, the average age at diagnosis was 68.5 years, and women were more predisposed to suffer from AM than men. About 26.4% of the patients had distant metastatis at the time of diagnosis. We also reported a higher proportion (41.4%) of rectal melanoma than previous studies. Moreover, the incidence of rectal melanoma was higher than that of anal melanoma between 2006 and 2011.

The estimated annual incidence of AM was 0.343 per 1 million, as well as 0.259 and 0.407 for male and female, respectively. The incidence increased with age and over time. Specifically, the incidence peaked in patients over 85-year old and in the period between 2006 and 2011, with incidence rates of 2.818 and 0.460, respectively. This finding is in agreement with the incidence data obtained from the North American Association of Central Cancer Registries; in this report, the incidence rate is 0.4 per million between 1996 and 2000 (based on 299 cases) and was age adjusted to the 2000 U.S. population standard.^19^ A total of 27 states and 1 metropolitan area participated in this study, and data covered more population than those of the SEER program. However, this program had no updated evaluation of the incidence of AM in the past decade. In Sweden, a population-based study covered about 95% cancer patients and covered 253 cases during a 40-year period (1960–1999).^20^ The reported incidence rates were 1.0 and 0.7 per million for female and male, respectively, and were significantly higher than those reported in the United States. Investigators in Australia also reported that the incidence of AM is 0.28 per million in 1985 to 1995, similar to the present results in the same period.^21^

We conducted univariate and multivariate analyses to predict the prognosis of AM. As predicted, stage was an independent factor of OS and CSS. Patients with local-stage AM showed improved survival than those with regional- and distant-stage AM. For the staging system of mucosal melanoma, the American Joint Committee on Cancer Tumor, Node, and Metastasis classification is used to stage mucosal melanoma of the head, neck, and vulva.^22^ However, a simplified clinical staging system was used to categorize vaginal and anorectal melanomas because of their rarity. Specifically, AM was classified based on disease distribution as stage I (local disease), stage II (regional nodal involvement), or stage III (distant metastasis).^4,23^ This finding is consistent with the SEER summary staging system adopted in the present study.

Surgical resection is the standard of care for AM, and patients undergoing surgery showed improved prognosis. In this research, patients with distant metastasis could not obtain significant survival benefits from the surgery. Hence, surgical resection may not be the optimal choice for distant-stage patients. With regard to the type of surgery, MEE with dissection of lymph nodes can control lymphatic spread and result in less local relapse; however, this technique confers long hospital stay, slow recovery, and a need for permanent stoma. In addition, a few studies reported that patients failed to achieve any survival benefits by using such an aggressive surgical approach.^5,10,24–26^ In 2010, Iddings et al^9^ analyzed 145 patients from the SEER database between 1973 and 2003 and concluded that the type of surgery did not affect OS or CSS. These findings are consistent with our results based on latest information and larger population. Therefore, if technically possible, we advise patients with AM to receive LEE to avoid unnecessary injuries and improve their quality of life.

However, this article still presents several limitations. First, as an SEER-based observational study, we were unable to get data regarding adjuvant therapy and detailed course of treatment, which are strongly associated with prognosis. Moreover, lack of information on comorbidity, recurrence, and treatment-related complications; migration of patients in and out of the SEER registry; and selection bias are factors that should be considered.^27,28^ Second, as patients were enrolled in SEER program from 1973 to 2011, the coding and staging system evolve and differ significantly over past 4 decades, which requires a thorough understanding of variables. Apparently, during such a long period, improvements in imaging and pathology may contribute to increased incidence rates, and improved chemotherapy and surgical techniques affect prognosis as well.^29,30^ Third, sample sizes were small in subgroup analyses, which may contribute to false positives or negatives. This limitation is inevitable for studies on such a rare tumor. Therefore, if possible, a prospective study with a large sample size must be performed in the future to validate the present results.

This population-based study provides an up-to-date estimation of the incidence and prognosis of AM by using the SEER data. The incidence of AM increased with age and over time. Tumor stage and surgery may be independent risk predictors, and patients with distant-stage AM could not obtain survival benefits from surgical treatment. Moreover, prognosis was not statistically different between LEE and MEE.

## Supplementary Material

Supplemental Digital Content
