# Supplementary material for: Incidence, Surgical Treatment, and Prognosis of Anorectal Melanoma From 1973 to 2011: A Population-Based SEER Analysis
Source: Medicine (Baltimore). 2016 Feb 18;95(7):e2770. doi: 10.1097/MD.0000000000002770 (PMC4998623; doi:10.1097/MD.0000000000002770)

| **Table S1. Characteristics of overall patients and included cases.** | | | | | | |
| --- | --- | --- | --- | --- | --- | --- |
|  | **Overall** | | | | **Included** ^a^ | **P Value** |
| **Total Patients (%)** | | | 640(100%) | | 485(75.8%) |  |
| **Age (SD)** | | |  | |  |  |
| Mean Age | | | 68.5±14.2 | | 67.5±14.8 | 0.237 ^b^ |
| Male | | | 65.9±14.1 | | 64.3±14.5 |  |
| Female | | | 70.1±14.0 | | 69.5±14.6 |  |
| **Gender (%)** | | |  | |  | 0.663 ^c^ |
| Male | | | 236(36.9%) | | 185(38.1%) |  |
| Female | | | 404(63.1%) | | 300(61.9%) |  |
| **Race (%)** | | |  | |  | 0.502 ^c^ |
| White | | | 537(83.9%) | | 396(81.6%) |  |
| Black | | | 40(6.3%) | | 33(6.8%) |  |
| Asian | | | 52(8.1%) | | 48(9.9%) |  |
| Others | | | 11(1.7%) | | 8(1.7%) |  |
| **Stage (%)** | | |  | |  | 0.400 ^c^ |
| Localized | | | 232(36.3%) | | 182(37.5%) |  |
| Regional | | | 165(25.8%) | | 105(21.6%) |  |
| Distant | | | 169(26.4%) | | 133(27.4%) |  |
| Unknown | | | 74(11.6%) | | 65(13.4%) |  |
| **Surgery (%)** | | |  | |  | 0.138 ^c^ |
| No surgery | | | 100(15.6%) | | 92(19.0%) |  |
| Surgery | | | 538(84.1%) | | 391(80.6%) |  |
| **Radiation (%)** | | | 123(19.2%) | | 103(21.2%) | 0.468 ^c^ |
| **Positive LNs Numbers (%)** | | | | |  | 0.388 ^c^ |
| 0 | | 75(11.7%) | | | 57(11.0%) |  |
| ≥1 | | 113(17.7%) | | | 86(17.7%) |  |
| No examination | | 375(58.6%) | | | 299(61.7%) |  |
| Unknown | | 77(12.0%) | | | 43(8.9%) |  |
| **Year of diagnosis (%)** | | | |  |  | 0.692 ^c^ |
| 1973-2000 | | 221(34.5%) | | | 173(35.7%) |  |
| 2001-2011 | | 419(65.5%) | | | 312(64.3%) |  |

Abbreviation: SD, standard deviation; LNs, lymph nodes.

^a^ Cases that were included for survival analysis

^b^ Independent-samples T test

^c^ Pearson Chi-square test

| **Table S2. Analysis of potential characteristics influencing cause specific survival.** | | | | | |
| --- | --- | --- | --- | --- | --- |
|  | **Univariate analysis** | | | **Multivariate analysis** | |
| **Variables** | **Median CSS (95%CI)** | | **P** | **HRs** | **P** |
| **Age at diagnosis** |  | |  |  |  |
| <=50 | 18(13.4-22.6) | |  |  |  |
| 51-70 | 16(13.3-18.7) | |  | NA |  |
| >=71 | 18(13.0-23.0) | | 0.816 |  |  |
| **Gender** |  | |  |  |  |
| Male | 18(12.6-23.4) | |  | NA |  |
| Female | 16(13.0-19.0) | | 0.374 |  |  |
| **Race** |  | |  |  |  |
| White | 16(13.3-18.7) | |  | NA |  |
| Non-white | 20(11.4-28.6) | | 0.591 |  |  |
| **Stage** |  | |  |  |  |
| Localized | 33(27.7-38.3) | |  | 1 |  |
| Regional | 20(13.9-26.1) | |  | 1.59(1.15-2.20) | 0.005 |
| Distant | 6(4.8-7.2) | | <0.001 | 2.98(2.11-4.21) | <0.001 |
| **Surgery** |  | |  |  |  |
| No surgery | 6(4.6-7.4) | |  | 1 |  |
| Surgery | 20(16.0-24.0) | | <0.001 | 0.69(0.47-1.00) | 0.048 |
| **Location** |  | |  |  |  |
| Anus | 22(17.5-26.5) | |  | 1 |  |
| Rectum | 11(8.3-13.7) | | 0.003 | 1.28(0.98-1.65) | 0.066 |
| **Year of diagnosis** |  | |  |  |  |
| 1973-2000 | | 22(14.1-29.9) |  | 1 |  |
| 2001-2011 | 15(12.1-17.9) | | 0.011 | 1.28(0.98-1.67) | 0.066 |

Abbreviations: CSS, cause specific survival; CI, confidence intervals; HRs, hazard ratios.

| **Table S3. Surgical treatment and cause specific survival by stratified analyses with year of diagnosis and stage.** | | | | | | | | | | |
| --- | --- | --- | --- | --- | --- | --- | --- | --- | --- | --- |
|  |  | **Surgery** | | | | **No Surgery** | | | |  |
|  | **Number (%)** | **Number (%)** | **Median CSS (95%CI)** | **3-Year CSS** | **5-Year CSS** | **Number (%)** | **Median CSS (95%CI)** | **3-Year CSS** | **5-Year CSS** | **P** |
| **Year of diagnosis** | |  |  |  |  |  |  |  |  |  |
| 1973-2000 | 173(35.7%) | 147(85.0%) | 27(17.3-36.7) | 0.32 | 0.24 | 26(15.0%) | 10(1.6-18.4) | 0.14 | 0.07 | 0.022 |
| 2001-2011 | 312(64.3%) | 244(78.2%) | 18(14.5-21.5) | 0.24 | 0.18 | 66(21.1%) | 4(2.7-5.3) | 0.10 | 0.10 | <0.001 |
| **Stage** |  |  |  |  |  |  |  |  |  |  |
| Localized | 182(37.5%) | 172(94.5%) | 33(25.1-40.9) | 0.41 | 0.27 | 10(5.5%) | 4(2.2-5.8) | 0 | 0 | 0.002 |
| Regional | 105(21.6%) | 100(95.2%) | 20(14.0-26.0) | 0.21 | 0.19 | 5(4.8%) | 3(0-11.8) | 0 | 0 | 0.021 |
| Distant | 133(27.4%) | 78(59.1%) | 7(3.8-10.2) | 0.08 | 0.06 | 54(40.9%) | 5(3.6-6.4) | 0.05 | 0.05 | 0.19 |

Abbreviations: CSS, cause specific survival; CI, confidence intervals.

|  | **Table S4. Subgroup analyses of surgery on CSS.** | | | | | | | | | | | | | |
| --- | --- | --- | --- | --- | --- | --- | --- | --- | --- | --- | --- | --- | --- | --- |
|  |  |  | | **LEE** | | | | | **MEE** | | | | |  |
|  |  | **Number (%)** | | **Number (%)** | **Median CSS (95%CI)** | **1-Year CSS** | **3-Year CSS** | **5-Year CSS** | **Number (%)** | **Median CSS (95%CI)** | **1-Year CSS** | **3-Year CSS** | **5-Year CSS** | **P**^a^ |
|  | **Rectum** |  | |  |  |  |  |  |  |  |  |  |  |  |
|  | **Year of diagnosis** | | |  |  |  |  |  |  |  |  |  |  |  |
|  | 1973-2000 | | 67(32.8%) | 25(37.3%) | 33(14.6-51.4) | 0.58 | 0.36 | 0.22 | 25(37.3%) | 13(0-32.2) | 0.46 | 0.31 | 0.31 | 0.327 |
|  | 2001-2011 | | 137(67.2%) | 63(46.0%) | 20(9.7-30.3) | 0.45 | 0.27 | 0.2 | 26(19.0%) | 12(9.8-14.2) | 0.14 | 0 | 0 | 0.269 |
|  | **Stage** | |  |  |  |  |  |  |  |  |  |  |  |  |
|  | Localized | | 76(27.0%) | 53(69.7%) | 32(17.9-46.1) | 0.67 | 0.38 | 0.22 | 13(17.1%) | 13(10.1-15.9) | 0.33 | 0.33 | 0.33 | 0.189 |
|  | Regional | | 34(12.1%) | 6(17.6%) | 7(4.9-9.1) | 0.20 | 0 | 0 | 24(70.6%) | 11(5.3-16.7) | 0.36 | 0.18 | 0.18 | 0.087 |
|  | Distant | | 65(31.9%) | 17(26.1%) | 7(4.0-10.0) | 0.20 | 0.20 | 0.20 | 12(18.5%) | 7(0.8-13.2) | 0.10 | 0 | 0 | 0.871 |
|  | **Anus** | |  |  |  |  |  |  |  |  |  |  |  |  |
|  | **Year of diagnosis** | | | | | | | | | | | | | |
|  | 1973-2000 | | 106(37.7%) | 58(54.7%) | 27(16.4-37.6) | 0.57 | 0.38 | 0.27 | 28(26.4%) | 16(12.6-19.4) | 0.35 | 0.23 | 0.16 | 0.795 |
|  | 2001-2011 | | 175(62.3%) | 94(53.7%) | 24(14.3-33.7) | 0.51 | 0.30 | 0.22 | 31(17.7%) | 18(7.6-28.4) | 0.48 | 0.28 | 0.13 | 0.523 |
|  | **Stage** | |  |  |  |  |  |  |  |  |  |  |  |  |
|  | Localized | | 106(37.7%) | 81(76.4%) | 33(29.4-36.6) | 0.66 | 0.41 | 0.26 | 14(13.2%) | 71(0-150.8) | 0.66 | 0.66 | 0.33 | 0.391 |
|  | Regional | | 71(25.3%) | 30(42.3%) | 23(16.8-29.2) | 0.48 | 0.28 | 0.28 | 26(36.6%) | 18(0-36.6) | 0.46 | 0.20 | 0.13 | 0.688 |
|  | Distant | | 68(24.2%) | 25(36.8%) | 10(4.7-15.3) | 0.21 | 0.07 | 0 | 16(23.5%) | 9(0-19.6) | 0.15 | 0.08 | 0 | 0.833 |

Abbreviations: CSS, cause specific survival; LEE, less extensive excision; MEE, more extensive excision; CI, confidence intervals.

^a^ Cox Regression method

**Figure S1.** Age-adjusted incidence per 1 million population per year in rectal and anal melanoma across nine age intervals.

**
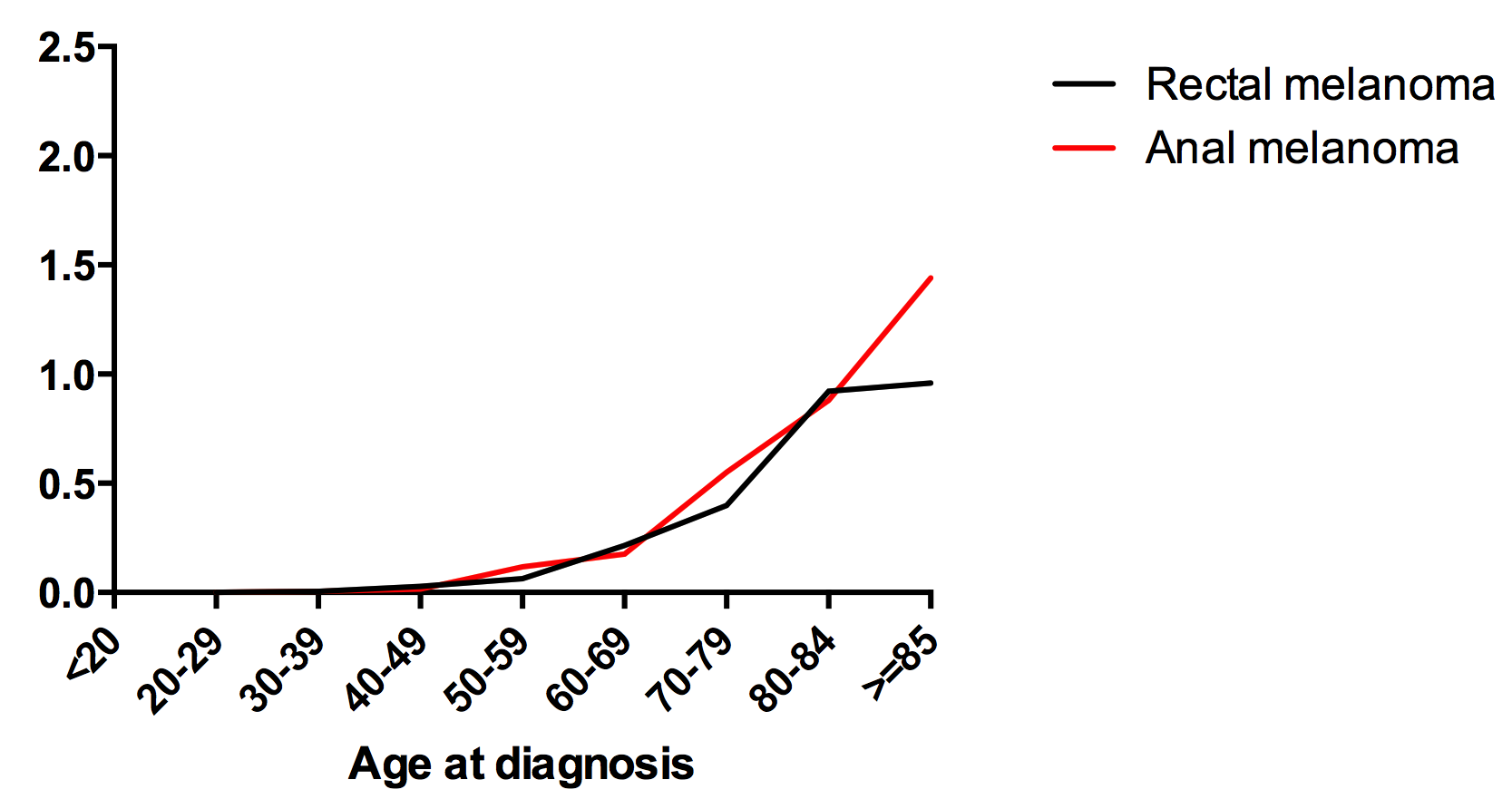
**

**Figure S2.** Age-adjusted incidence per 1 million population per year in rectal and anal melanoma over study periods.


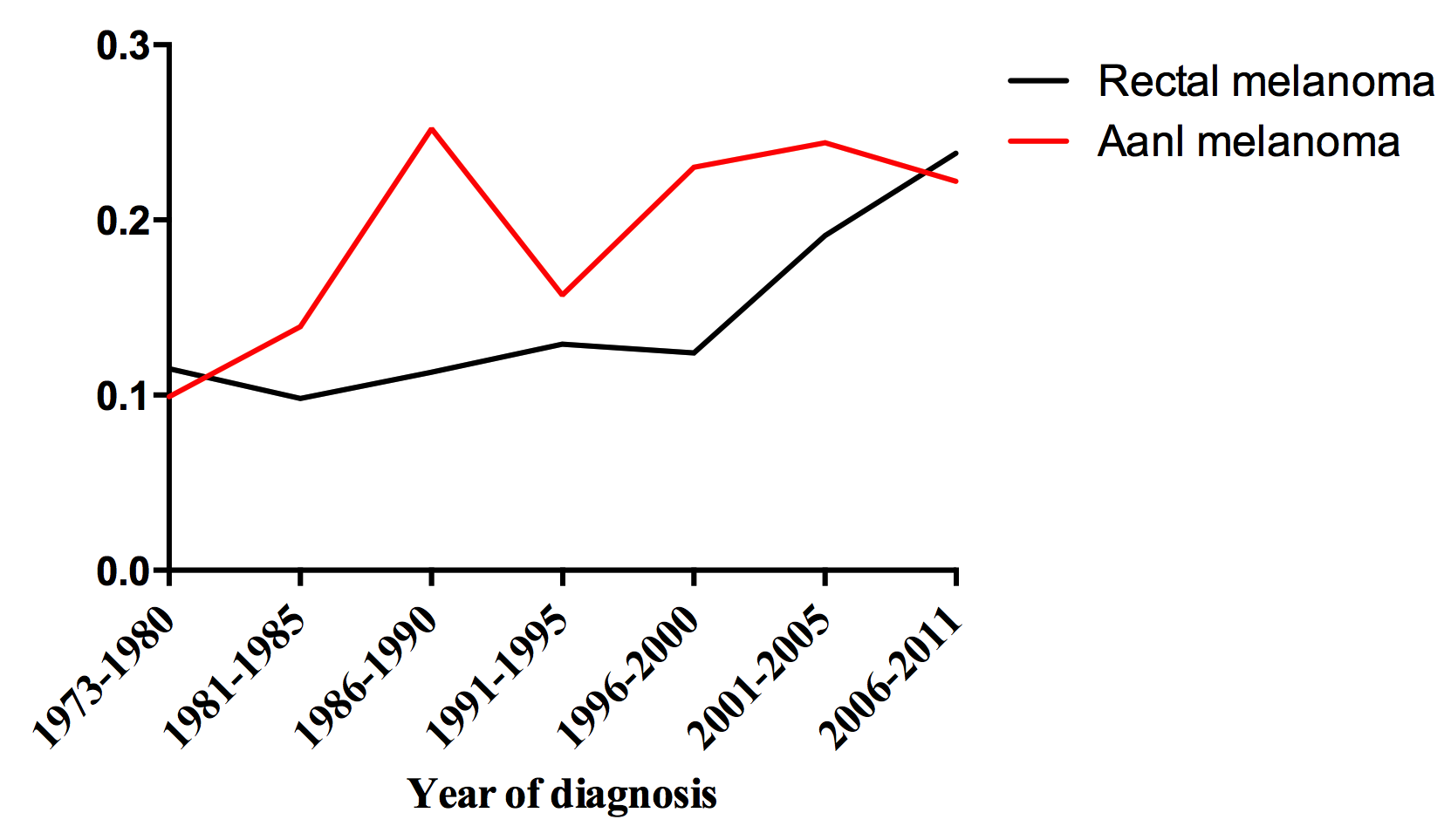

Supplement: Supplemental Digital Content [file medi-95-e2770-s001.doc]
